# Management of Agitation Following Aneurysmal Subarachnoid Hemorrhage: Is There a Role for Beta-Blockers?

**DOI:** 10.1155/2012/753934

**Published:** 2012-05-17

**Authors:** Fayaz Ibrahim, Ramaswamy Viswanathan

**Affiliations:** ^1^Kaweah Delta Health Care District, 1100 S Akers Street, Visalia, CA 93277, USA; ^2^SUNY Downstate Medical Center, Brooklyn, NY 11203, USA

## Abstract

*Introduction*. Stroke is a leading cause of mortality and morbidity in the United States. About 20% of the stroke is hemorrhagic and about 50% of these is due to aneurysmal subarachnoid hemorrhage. A troublesome neuropsychiatric complication of subarachnoid hemorrhage is agitation/aggression. *Case Presentation*. A 45-year-old man with no prior psychiatric history, sustained subarachnoid hemorrhage. After initial stabilization for 2 days, he underwent craniotomy and clipping of anterior cerebral communicating artery aneurysm. Treatment was continued with labetalol, nimodipine, and levetiracetam. Beginning postoperative day 4, patient developed episodes of confusion and agitation/aggression. Switching of Levetiracetam to valproate did not show any improvement. Psychiatry team tried to manage him with intense nursing intervention and different medications like olanzapine, valproate, lorazepam, and haloperidol. However, patient continued to be agitated and aggressive. Switching from labetalol to metoprolol resulted in dramatic improvement within 3 days. *Discussion*. Antipsychotics and benzodiazepines are often not sufficiently effective in the control of agitation/aggression in patients with traumatic brain injury and similar conditions. Our case report and the literature review including a cochrane review suggests that beta-blockers may be helpful in this situation.

## 1. Introduction

Stroke is a leading cause of mortality and morbidity in the United States (USA). About 20% of the stroke is hemorrhagic and about 50% of these is due to aneurysmal subarachnoid hemorrhage [1]. Approximately 85% of these occurs in the anterior circulation of the circle of Willis, thus leading to involvement of frontal lobe and subsequent behavioral problems. Though the recent advances in neurosurgical techniques have significantly reduced mortality, a significant percentage of them develop both short-term and long-term neurocognitive impairment. Neurobehavioral sequelae seem to be the most debilitating both to patients and their care givers and includes cognitive, psychiatric disorders, and agitation/aggression.

There is little doubt that acquired brain injury causes agitated and aggressive behavior. However, it is difficult to accurately quantify the problem as there is no consensus on the definition of agitation or aggression and these terms are often used interchangeably. Studies report a range of 11% to 33.7% incidence for agitation and aggression following brain injury [2, 3]. Acquired brain injury (ABI) is as such a heterogenous group; premorbid brain pathology, nature of the injury, and timing of surgical intervention are just some of the factors which determines outcome.

## 2. Case Description

A 45-year-old man with no prior psychiatric history was brought to the emergency room for altered mental status and after initial assessment and work up, he was diagnosed with subarachnoid hemorrhage (SAH). The patient was hemodynamically stabilized for the next 2 days following which he underwent craniotomy and clipping of anterior cerebral communicating artery aneurysm. Following surgery, treatment was continued with labetalol to control elevated blood pressure, nimodipine to counteract cerebral vasospasm, and levetiracetam for seizure prophylaxis.

Beginning postoperative day 4, the patient developed episodes of explosive outbursts of agitation and aggression. Patient was initially given lorazepam 2 mg IV Q 6 hrs by the primary team and a neurology consultation was requested concurrently. Neurologists noted that the CNS exam was unremarkable, but they expressed concern regarding possible worsening of agitated/aggressive behavior with use of levetiracetam. Based on the neurologist's recommendation, levetiracetam was switched to valproate sodium, starting with loading dose of 1000 mg IVPB, followed by 500 mg IVPB Q 12 hrs.

Although the patient did not develop any seizure activity, his agitated behavior continued to be problematic. Three days after the switch, he continued to display episodic agitated behavior. At this stage the primary team requested psychiatry consultation for pharmaceutical management of agitation. The psychiatry team tried to manage the agitation/aggression with intense nursing intervention and various medications such as olanzapine (up to 10 mg IM Q 12 hrs), valproate (up to 1000 mg daily), lorazepam (2 mg IM Q 6 hrs), and haloperidol (10 mg IM Q 8 hrs).

However, all the interventions were unsuccessful, and the patient continued to remain agitated and aggressive.

We then considered beta blockers as a possible treatment for uncontrolled agitated and aggressive behavior. Although he was already on labetalol for the treatment of hypertension, since labetalol is hydrophilic and does not readily cross the blood brain barrier, we decided to switch to a lipophilic beta-blocker. We initiated treatment with metoprolol succinate at 50 mg po daily with close monitoring of his hemodynamic status (blood pressure and heart rate). The next day it was increased to 100 mg po daily. Within 3 days, the patient demonstrated significant decrease in agitation and aggression. The benefits were sustained up to a follow-up period of 2 weeks, and the patient was successfully discharged back to the community.

## 3. Discussion

Although many medications such as SSRIs, valproate, lithium, haloperidol, TCAs, and buspirone are recommended, there is none approved by the FDA for treatment of agitation and aggressive behavior following ABI. Researchers conducted a survey among 129 physicians divided into experts or nonexperts, to study the prescription practices of physicians [4]. Experts either had published ≥2 articles on pharmacological interventions for ABI in the last 5 year, or had 70% of their practice devoted to treating ABI. Surprisingly experts most frequently prescribed carbamazepine, beta blockers, and TCAs. Nonexperts chose haloperidol four times more frequently than experts. Clearly there is no consensus among prescribers. A neurobehavioral guidelines working group conducted extensive literature review and identified the dearth of well-controlled studies in this area. The group also suggested that beta blockers may be beneficial [5].

Researchers studied 148 patients with SAH who were randomly assigned to receive just standard care or standard care with adrenergic blocking agents (propranolol with or without phentolamine) for 3 weeks [6]. Significantly lesser neurological deficits up to one-year period particularly in women was noted in the latter group. The study attributed benefits to beta blockade rather than alpha blockade. A Cochrane review published in 2008 included 6 RCTs of which 4 evaluated beta blockers propranolol and pindolol [7]. The study concluded “Firm evidence that carbamazepine or valproate is effective in the management of agitation and/or aggression following ABI is lacking…. Beta blockers have the best evidence for efficacy and deserve more attention”. Although most studies used nonselective beta blockers (ex; propranolol), selective beta blockers can also be useful. It was thought that beta-2 blockade was necessary for antiaggressive properties, but there are case reports of successful use of beta 1 selective blocker metoprolol in intermittent explosive disorder and mental retardation [8, 9].

Beta blockers have been shown to have benefits in treating some of the psychiatric conditions such as psychogenic polydipsia, antipsychotic-induced akathisia, and posttraumatic stress disorder, but it is unclear how they mediate these effects [10]. Several hypotheses have been postulated but there are no conclusive data. Some studies report benefits with beta blockers that do not cross the blood-brain barrier [11], thus suggesting that both central and peripheral mechanisms are involved. Specifically in a case of SAH, arterial spasm leads to significant complications due to cerebral hypoxia.

## 4. Conclusion

Antipsychotics and benzodiazepines are often not sufficiently effective in the control of agitation/aggression in patients with organic brain injury. Most ABI patients are sensitive to delirious side effects of medication which in turn can worsen the agitated behavior. They are also at increased risk for neuroleptic malignant syndrome and tardive dyskinesia. Our case report and the literature suggest that beta blockers may be helpful in this situation. They can treat concurrent hypertension and tachycardia that often accompany SAH. They cause less sedation compared to tranquillizers, thereby interfering less with clinicians' daily monitoring of the patient's clinical condition. Our case illustrates that beta-1 selective blockers can be beneficial even at low doses in treating uncontrolled aggression following SAH. It may be extremely useful in patients with chronic obstructive pulmonary disease where risk of bronchoconstriction may prevent use of nonselective beta blockers.
